# An Attempt to Polarize Human Neutrophils Toward N1 and N2 Phenotypes *in vitro*

**DOI:** 10.3389/fimmu.2020.00532

**Published:** 2020-04-28

**Authors:** Mareike Ohms, Sonja Möller, Tamás Laskay

**Affiliations:** Department of Infectious Diseases and Microbiology, University of Lübeck, Lübeck, Germany

**Keywords:** neutrophils, tumor-associated neutrophils, N1, N2, polarization, neutrophil heterogeneity, *Leishmania donovani*, visceral leishmaniasis

## Abstract

Neutrophils act as the first line of defense against invading pathogens. Although traditionally considered in context of their antimicrobial effector functions, the importance of tumor-associated neutrophils (TANs) in the development of cancer has become increasingly clear during the last decade. With regard to their high plasticity, neutrophils were shown to acquire an anti-tumorigenic N1 or a pro-tumorigenic N2 phenotype. Despite the urgent need to get a comprehensive understanding of the interaction of TANs with their tumor microenvironment, most studies still rely on murine tumor models. Here we present for the first time a polarization attempt to generate N1 and N2 neutrophils from primary human neutrophils *in vitro*. Our results underscore that N1-polarized neutrophils have a pro-inflammatory phenotype characterized among others by a higher level of intercellular adhesion molecule (ICAM)-1 and high secretion of interferon (IFN)γ-induced protein 10 (IP-10)/C-X-C motif chemokine 10 (CXCL10) and tumor necrosis factor (TNF). Further, we demonstrate that neutrophils incubated under a tumor-mimicking *in vitro* environment show a high cell surface expression of C-X-C motif chemokine receptor 2 (CXCR2) and secrete high levels of interleukin (IL)-8. These findings suggest that it is feasible to polarize blood-derived primary human neutrophils toward N1- and N2-like phenotypes *in vitro*. Further, we hypothesized that the presence of anti-inflammatory neutrophil phenotype is not a phenomenon limited to cancer but also occurs when neutrophils are infected with intracellular pathogens. Indeed, our findings indicate that N2-polarized neutrophils exert a markedly decreased capacity to kill the protozoan parasite *Leishmania donovani* and therefore permit parasite persistence.

## Introduction

Neutrophils, also known as polymorphonuclear leukocytes (PMN), are the predominant immune cell population in human blood. Neutrophils are produced in large numbers, ∼10^11^ cells per day, in the bone marrow from hematopoietic stem cells during granulopoiesis (1). They are regarded as the body’s first line of defense against invading pathogens, which they fight by using an array of diverse antimicrobial effector mechanisms ranging from phagocytosis, the release of granules to the formation of NETs. In recent years, however, accumulating evidence has shown that neutrophils possess greater functional diversity than previously appreciated and appear to play an important role under non-infectious conditions as well, most notably in cancer. Apart from cancer cells, solid tumors contain often different types of immune cells, including infiltrating neutrophils (2). The neutrophils found in the tumor are referred to as TANs. The mobilization of neutrophils from the circulation toward the tumor is controlled by the C-X-C motif chemokine receptor 2 (CXCR2) axis (3). Various cell types in the tumor release CXCR2 ligands, such as C-X-C motif chemokine (CXCL)1–3 and CXCL5–8 (4). Neutrophils expressing CXCR2 on their surface sense the chemotactic gradient move toward the higher concentration of CXCR2 ligands and get in direct contact with the tumor. Neutrophils display a high plasticity with the ability to adapt their function to the needs of their surrounding micromilieu. Several factors in the tumor microenvironment influence the polarization of recruited neutrophils toward distinct phenotypes. Based on their polarization status, two populations of TANs were recognized, the anti-tumorigenic N1 and the pro-tumorigenic N2 phenotype. This nomenclature was first proposed by Fridlender et al. in 2009 (5) based on similarity to TAMs and the terms M1 and M2. N1 and N2 populations of neutrophils are primarily defined by their functional phenotype because no specific cell surface markers have been identified so far for N1 and N2 neutrophils. N1 neutrophils exhibit an increased tumor cell cytotoxicity and an immunoactivating ability by the elevated production of TNF, intercellular adhesion molecule (ICAM)-1, ROS, and Fas (CD95), as well as the property to decrease the arginase expression (5, 6). In contrast, N2 neutrophils are characterized by higher expression of pro-tumor factors supporting tumor growth (5, 7). Further, in murine tumors, both populations can be distinguished morphologically as N1 neutrophils have hypersegmented nuclei in contrast to the N2 neutrophils, which are circular (5, 8). From a classical view, neutrophils are fully differentiated cells with a short life span. However, TANs display a remarkable longevity (9), and N1 and N2 TANs can possibly be converted into each other. The exposure of neutrophils to transforming growth factor β (TGF-β) (5, 7, 10) or granulocyte colony-stimulating factor (G-CSF) (11) was shown to induce the polarization toward the N2 phenotype. In contrast, a blockade of the TGF-β signaling or a type I interferon (IFN) treatment (12) results in N1 polarization *in vivo*.

The occurrence of opposing neutrophil subpopulations driving disease progression is not limited to cancer. Pro- and anti-inflammatory populations of neutrophils were further observed in myocardial infarction (13), SLE, rheumatoid arthritis or rheumatic fever (14), sepsis (15), psoriasis (16), HIV infection (17), asthma (18), antineutrophil cytoplasmic antibody (ANCA)-associated vasculitis (19), malaria (20), and Chagas’ disease (21). The consideration of neutrophil subpopulations in infection and inflammation might explain the exacerbation of certain pathologies if one neutrophil phenotype gets prevalent. An example for the contribution of neutrophils to disease outcome is the infection with the intracellular protozoan parasite *Leishmania*. Neutrophils possess direct antileishmanial capacities (22). However, as evidenced by parasite persistence (23) and abuse of neutrophils as “Trojan horses” to silently enter macrophages (24, 25), direct parasite killing by neutrophils is inefficient. Several similarities between TANs and the neutrophils from VL patients could be observed. During the acute phase of VL, neutrophils show increased expression of the immunosuppressive molecules interleukin (IL-10) and arginase (26). Further, *Leishmania* infection was reported to extend the life span of neutrophils up to 2–3 days (27). Considering the likelihood of a polarization of neutrophils toward an immunosuppressive phenotype during *L. donovani* infection, we hypothesize here that in VL, *L. donovani* can induce the polarization of neutrophils toward an anti-inflammatory and non-antimicrobial N2 phenotype in order to survive inside neutrophils.

Despite the enormous interest in TANs in the recent years, our current understanding of the role of neutrophils in tumor development is primarily based on murine models of cancer. To our knowledge, this is the first polarization attempt to polarize human N1 and N2 neutrophils *in vitro*. In this experimental setup, we cultivated primary human neutrophils in the presence of a pro-inflammatory polarization cocktail to generate N1 neutrophils, whereas N2 neutrophils were generated by incubation with a polarization cocktail mimicking hallmarks of the tumor microenvironment. To evaluate whether the neutrophil phenotypes generated in our *in vitro* study share characteristics with *in vivo* polarized neutrophils, phenotypic markers and features described for N1 or N2 TANs were assessed. In addition, as a measure of antimicrobial capacity, the anti-leishmanial capacity of the *in vitro* polarized neutrophils was determined.

## Materials and Methods

### Ethics Statement

Blood collection was conducted with the agreement and written consent form of each participant and was approved by the Ethical Committee of the University of Lübeck (18–186).

### Isolation of Primary Human Neutrophils

Peripheral blood was collected by venipuncture from healthy adult volunteers using lithium–heparin collection tubes (S-Monovette^®^ 9 ml LH, Sarstedt, Nümbrecht, Germany). Blood was layered on a two-layer density gradient consisting of an upper layer of Histopaque^®^ 1077 (Sigma Aldrich, Steinheim, Germany) and a lower layer of Histopaque^®^ 1119 (Sigma Aldrich) and centrifuged for 5 min at 300 × *g* followed by 25 min at 800 × *g*. Cells from the upper layer consisting mainly of lymphocytes and monocytes were discarded. The granulocyte-rich lower layer was collected, leaving the erythrocyte pellet at the bottom of the tube. Granulocytes were washed once in 1 × Dulbecco’s phosphate-buffered saline (DPBS) (Thermo Fisher, Germany) for 10 min at 800 × *g*, resuspended in complete medium [RPMI 1640 Medium (Sigma Aldrich) supplemented with 2 mM L-glutamine (Merck, Darmstadt, Germany), 10 mM 4-(2-hydroxyethyl)-1-piperazineethanesulfonic acid (HEPES; Life Technologies, Darmstadt, Germany), 10% heat-inactivated (FCS; Gibco, Germany), 100 U/ml penicillin, and 100 μg/ml streptomycin (Biochrom, Berlin, Germany)] and further fractionated on a discontinuous Percoll^®^ (GE Healthcare, Braunschweig, Germany) gradient consisting of layers with densities of 1.105 g/ml (85%), 1.100 g/ml (80%), 1.087 g/ml (70%), and 1.081 g/ml (65%). After centrifugation for 25 min at 800 × *g*, the interface between the 80% and 70% Percoll^®^ layers was collected. The cells were washed once in 1× DPBS for 10 min at 800 × *g* and resuspended in complete medium to a concentration of 5 × 10^6^ cells/ml. All described procedures were conducted at room temperature and under sterile conditions. Cell counting was conducted with a hemocytometer (Imp. Neubauer, 0.0025 mm^2^, depth 0.100 mm, VWR, Dresden, Germany) and crystal violet staining. The preparations contained ≥99% granulocytes (Supplementary Figure S1), of which >95% were neutrophils and 1%–4% were eosinophils, as determined by Giemsa staining (Diff Quik Fix, Medion Diagnostics, Berlin, Germany) of cytocentrifuged (Shandon) samples. Contamination with monocytes, T and B lymphocytes was lower than 1% (Supplementary Figure S2).

### *In vitro* Culture and Polarization of Neutrophils

Polarization of neutrophils toward an N1-like phenotype was conducted in six-well plates (Greiner, Kremsmünster, Austria) with 3 ml/well at a cell concentration of 5 × 10^6^/ml in complete medium supplemented with an N1 polarization cocktail containing 100 ng/ml lipopolysaccharide (LPS; Sigma Aldrich), 50 ng/ml IFNγ (R&D Systems, Wiesbaden, Germany), and 10,000 U/ml IFNβ (R&D Systems) at 37°C in a humidified air atmosphere containing 5% carbon dioxide (CO_2_). Polarization of neutrophils toward an N2-like phenotype was conducted in six-well plates (Greiner) with 3 ml/well in complete medium supplemented with an N2 polarization cocktail containing 25 mM L-lactate (Sigma Aldrich), 10 μM adenosine (Merck), 20 ng/ml TGF-β (PeproTech, Hamburg, Germany), 10 ng/ml IL-10 (BioLegend, San Diego, CA), 20 ng/ml prostaglandin E2 (PGE_2_; Tocris, Bristol, United Kingdom), and 100 ng/ml granulocyte colony-stimulating factor (G-CSF; PeproTech). The pH of the medium containing the N2 polarization cocktail was adjusted to 6.7 with hydrochloric acid (HCl; Roth, Carlsruhe, Germany) and sodium hydroxide (NaOH; Merck). Cultivation of N2-like neutrophils was conducted in a humidified hypoxic chamber (Toepffer Lab Systems, Göppingen, Germany) with 2% oxygen (O_2_) and 5% CO_2_ at 37°C. Since neutrophils have a short life span and undergo apoptosis within a few hours, the cultivation of neutrophils in the polarization experiments was carried out in the presence of 3 μM pan caspase inhibitor QVD-Oph (R&D Systems). This treatment inhibits spontaneous neutrophil apoptosis and, consequently, enhances its life span. In the polarization experiments, the control cells were cultivated in complete medium containing 3 μM QVD-OPh. These control cells were named N0. All polarization experiments were conducted at 37°C in a humidified air atmosphere containing 5% CO_2_. Functions and phenotypes of the cells were assessed 24 and 48 h after incubation in the given polarization cocktails (N1 or N2) or without polarizing agents (N0).

### Flow Cytometry of Cell Surface Molecules

Polarized neutrophils (500,000 cells/sample) were resuspended in FACS buffer [1 × PBS (Thermo Fisher) supplemented with 1% bovine serum albumin (Albumin fraction V, Roth), 1% human serum, and 0.01% sodium azide (Sigma Aldrich)] in a V-bottom plate. After washing once with FACS buffer, the cells were stained with Pacific Blue^TM^-conjugated mAb to human CD95 [clone DX2, immunoglobulin G1 (IgG1), Biolegend, San Diego, CA], allophycocyanin (APC)-conjugated mAb to human CD54 (clone HA58, IgG1, Biolegend), APC-conjugated mAb to human CD182 (clone 5E8/CXCr2, IgG1, Biolegend), fluorescein isothiocyanate (FITC)-conjugated mAb to human CD66b (clone G10F5, IgM, BD, Heidelberg, Germany), APC-conjugated mAb to human CD62L (clone DREG-56, IgG1, Biolegend), and phycoerythrin (PE)-conjugated mAb to human CD11b (clone 2LPM19c, IgG1, Dako, Waldbronn, Germany) for 30 min at 4°C protected from light.

After two wash steps with FACS buffer at 4°C, the cells were resuspended in FACS buffer and measured with a BD FACS Canto II (BD) flow cytometer. Data analysis was conducted with FlowJo V10.0.7. Representative FACS plots showing gating strategy (Supplementary Figure S2A), percentage of cells or mean fluorescence intensity (MFI) are shown in Supplementary Figure S3 (CD66b, CD11b), Supplementary Figure S4 (CD62L), and Supplementary Figure S5 (CD95, CD54, CD182).

### Determination of Cytokines in Culture Supernatants

Cell-free supernatants of polarized neutrophils were collected and stored at −20°C until cytokine determination. The ELISAs for TNF, IFNγ-induced protein 10 (IP-10), myeloperoxidase (MPO), and IL-8 (all DuoSet^®^ ELISA from R&D Systems) were conducted in accordance to the manufacturer’s instructions.

### Detection of Reactive Oxygen Species

The sum of intra- and extracellular MPO-derived ROS (28) was measured by using a luminol-amplified chemiluminescence assay. Polarized neutrophils (400,000 cells/sample) were resuspended in complete medium without FCS and seeded in a flat-bottom white 96-well plate (Nunc^TM^ F96 MicroWell^TM^ polystyrol plate, Thermo Fisher). Subsequently, 60 μM luminol (Invitrogen, Germany) was added, and the cells were stimulated by the addition of 20 nM phorbol 12-myristate 13-acetate (PMA; Sigma Aldrich). The chemiluminescence resulting from ROS release was analyzed immediately by an Infinite M200pro-Tecan reader (Tecan, Männedorf, Switzerland) and Tecan i-control 1.7 software. ROS release was monitored every minute for a period of 1 h at 37°C and 5% CO_2_. Extracellular superoxide was detected by using a lucigenin-amplified chemiluminescence assay (28). This assay was performed the same way as the luminol assay, but with 0.2 mM lucigenin (Alexis, Loerrach, Germany) instead of luminol.

### *Leishmania donovani* Culture

Virulent *L. donovani* promastigotes (strain MHOM/IN/82/Patna 1) were cultivated in Schneider’s *Drosophila* Medium with L-glutamine (Genaxxon, Ulm, Germany) supplemented with 10% FCS, 100 U/ml penicillin, 100 μg/ml streptomycin, and 2% sterile filtered human urine at 27°C in a humidified air atmosphere containing 5% CO_2_. For seeding of cultures, a parasite density of 1 × 10^6^ cells/ml was used. The parasite counting was conducted in a hemocytometer with a chamber depth of 0.02 mm (0.0025 mm^2^, depth 0.02 mm, VWR). The culture was considered to be in the stationary phase 72 h after seeding. Serial passaging was conducted until passage 10.

### *In vitro* Infection of Neutrophils With *Leishmania donovani* Promastigotes

Neutrophils and *L. donovani* promastigotes were resuspended in complete medium and coincubated at a parasite–neutrophil ratio of 10:1 and a neutrophil density of 5 × 10^6^ cells/ml for 3 h at 37°C in a humidified air atmosphere containing 5% CO_2_. Subsequently, the infection rate was assessed on Giemsa-stained cytocentrifuge samples. The cells were washed six times in 1 × PBS at 400 × *g* for 10 min to remove extracellular *Leishmania*. Infected cells were again adjusted to 5 × 10^6^ cells/ml.

### Quantification of *Leishmania donovani* Survival in Polarized Neutrophils

A limiting dilution culture assay was used to detect viable *L*. *donovani* parasites in polarized neutrophils as described previously (29). Briefly, serial 1.5-fold dilutions of *L*. *donovani*-infected neutrophil suspensions (5 × 10^6^ cells/ml) were plated in four replicates in 96-well flat-bottom microtiter plates containing Schneider’s *Drosophila* Medium. The plates were incubated at 27°C in humidified air atmosphere containing 5% CO_2_ for 10–14 days. The growth of *L*. *donovani* promastigotes was detected microscopically. The last dilution resulting in a growth of parasites in >50% of the wells is given as a quantitative measure of the parasite load in the neutrophil cell suspension.

### Statistical Analysis

If not stated differently, the presented data were generated from a minimum of three independent experiments with neutrophils isolated from different blood donors. Statistical analysis was performed with the GraphPad Prism software 6 using the one-way ANOVA followed by Sidak’s *t*-test for multiple comparisons or unpaired *t*-test followed by Welch’s correction. A *p*-value ≤ 0.05 was considered statistically significant.

## Results

### *In vitro* Polarized Neutrophils Express Characteristic Surface Markers of N1 and N2 Neutrophils

The N1/N2 terminology of tumor-infiltrating neutrophils was established in analogy of tumor infiltrating M1 and M2 macrophages (5). M1 macrophages activated by IFNs and bacterial products can elicit tumor-destructive reactions by targeting tumor cells (30). The alternatively activated M2 macrophages, on the other hand, support tumor progression by affecting cancer cell proliferation and survival, angiogenesis, and response to hormones (31). Accordingly, in our *in vitro* approach, the N1 polarization cocktail was composed of lipopolysaccharide (LPS), IFNγ, and IFNβ (Table 1). IFNγ and the bacterial product LPS are known to activate neutrophil functions (32, 33). IFNβ was shown to be an important factor for the generation of N1 TANs (12).

**TABLE 1:** Composition of N1 and N2 polarization cocktails.

	Substance	References
N1	LPS	(32)
	IFNγ	(33)
	IFNβ	(12)
N2	TGFβ	(5)
	IL-10	(52, 53)
	PGE_2_	(54, 55)
	G-CSF	(56–58)
	L-lactate	(59)
	Adenosine	(60)

*The cited references refer to literature describing the effects of the substances on neutrophils regarding N1/N2 functions and/or polarization. G-CSF, granulocyte colony-stimulating factor; IFN, interferon; IL, interleukin; LPS, lipopolysaccharide; PGE_2_, prostaglandin E2; TGF, transforming growth factor.*

The term N2-phenotype for tumor-promoting neutrophils is based on the functional similarity to tumor-infiltrating M2 macrophages (5). The alternatively activated M2 macrophages support tumor progression by affecting cancer cell proliferation and survival, angiogenesis, and response to hormones (31). To create the N2 polarization cocktail, we added substances that were reported previously in *in vivo* studies to be associated with the generation of N2 TANs (Table 1). Accordingly, the N2 polarization cocktail contained TGF-β, IL-10, PGE_2_, and G-CSF. Since high levels of L-lactate and adenosine are characteristic for a tumor microenvironment, L-lactate and adenosine were added to the N2 polarization cocktail (Table 1). In order to further mimic the tumor environment, polarization toward the N2 phenotype was carried out at a low pH and under low oxygen conditions that are additional characteristic features for the tumor environment. The pH of the medium for the N2 polarization was adjusted to 6.7, and the cells were incubated under hypoxic conditions with 2% oxygen. Primary human neutrophils from healthy donors were incubated for 24 or 48 h in the presence of an N1 or N2 polarization cocktail. As polarization effects were investigated over a longer time period, the cells were treated with the Pan-caspase inhibitor QVD-OPh to inhibit spontaneous apoptosis and, consequently, to prolong their life span (34). Treatment with QVD-OPh efficiently suppressed spontaneous apoptosis (Supplementary Figure S6) and cell death (Supplementary Figures S6, S7) of primary human neutrophils for up to 48 h but did not lead to activation (Supplementary Figure S8) or ROS production (Supplementary Figure S9). Neutrophils treated with QVD-OPh in complete medium served in every experiment as control and were further termed N0.

**FIGURE 1:**
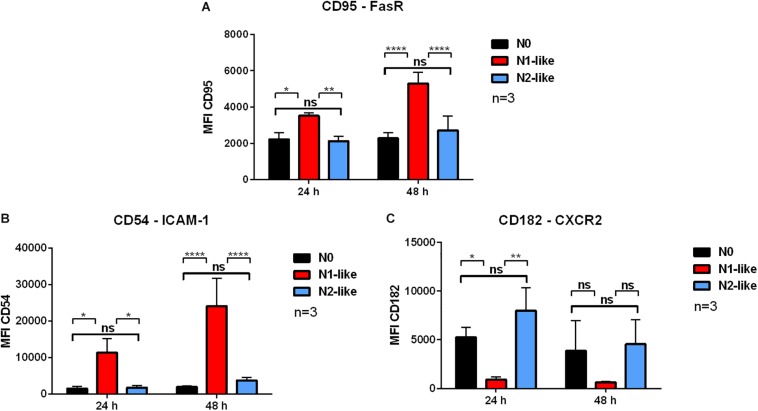
Cell surface expression of typical N1 and N2 markers on *in vitro* polarized neutrophils. Primary human neutrophils were incubated for 24 or 48 h in the presence of an N1 or N2 polarization cocktail containing the Pan-caspase inhibitor QVD-OPh. Neutrophils which were only treated with QVD-OPh (N0) served as control. The cell surface expression of the typical N1 markers FasR (CD95) **(A)** and intercellular adhesion molecule (ICAM)-1 (CD54) **(B)**, and the typical N2 marker C-X-C motif chemokine receptor 2 (CXCR2) (CD182) **(C)** was assessed by using flow cytometry. Bar diagrams show mean fluorescence intensity (MFI) ± SD (*n* = 3). *p < 0.05, **p < 0.01, ****p < 0.0001, ns = not significant.

To test whether the *in vitro* polarized neutrophils correspond to the N1 and N2 phenotypes observed *in vivo*, several typical N1 and N2 surface markers (Table 2) were investigated by flow cytometry. These included FasR (CD95) and ICAM-1 (CD54) as typical N1 markers (Table 2). Both markers were significantly increased in neutrophils cultured with the N1 polarization cocktail compared to N0 and N2-like neutrophils (Figures 1A,B and Supplementary Figure S5). As N2 marker, the cell surface expression of CXCR2 (CD182) (Table 2) was assessed. The expression of receptor CXCR2 was significantly increased in N2-like as compared to N1-like neutrophils at least after 24 h (Figure 1C and Supplementary Figure S5).

**TABLE 2:** Phenotypic features used as readout markers for the characterization of *in vitro* polarized neutrophils.

N1	N2	References	Assay
			technique
CD54^high^	CD54^low^	(3)	Flow cytometry
CD95^high^	CD95^low^	(3)	Flow cytometry
CD182^low^	CD182^high^	(61, 62)	Flow cytometry
TNF production high	TNF production low	(5)	ELISA
IP-10 production high	IP-10 production low	(5)	ELISA
IL-8 production low	IL-8 production high	(3)	ELISA
H_2_O_2_ production high	H_2_O_2_ production low	(6)	Luminol assay

*The cited references refer to literature describing the differences between N1 and N2 TANs in murine tumor models. H_2_O_2_, hydrogen peroxide; IL, interleukin; IP-10, interferon-γ-induced protein 10; TANs, tumor-associated neutrophils; TNF, tumor necrosis factor.*

### *In vitro-*Generated N1-Like Neutrophils Are Highly Activated

**FIGURE 2:**
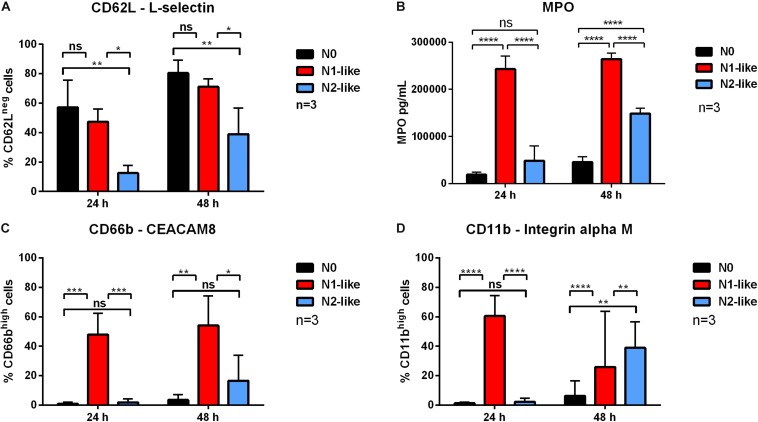
Cell surface expression of the activation marker CD62L and of the degranulation markers CEACAM8 (CD66b), integrin alpha M (CD11b), as well as secretion of myeloperoxidase (MPO) by *in vitro* polarized neutrophils. Primary human neutrophils were incubated for 24 and 48 h in the presence of an N1 or N2 polarization cocktail including the Pan-caspase inhibitor QVD-OPh. The cell surface expression of CD62L **(A)**, CD66b **(C)**, and CD11b **(D)** was assessed by using flow cytometry. For the detection of MPO **(B)**, cell-free supernatants were collected after 24 and 48 h. The concentration of MPO was measured by ELISA. Bar diagrams show mean fluorescence intensity (MFI) ± SD (*n* = 3). *p < 0.05, **p < 0.01, ***p < 0.001, ****p < 0.0001, ns = not significant.

To obtain information about the activation status of the polarized cells, the activation marker L-selectin (CD62L) as well as the degranulation markers CEACAM8 (CD66b) and integrin alpha M (CD11b) were assessed *via* flow cytometry. Since activation of neutrophils results in the shedding of cell surface L-selectin (35), this cell surface molecule is a widely used marker to distinguish CD62L^*neg*/*low*^ activated cells from CD62L^*high*^ non-activated neutrophils. Both CD66b and CD11b are present in the membrane of neutrophil granules. Since upon degranulation, the granule membranes fuse with the cell membrane, the enhanced cell surface presence of CD66b and CD11b is a reliable indirect marker of neutrophil degranulation. In addition, degranulation was assessed by measurement of secreted MPO in the supernatants of polarized cells. As compared to N0 and N1-polarized cells, a significantly reduced shedding of CD62L was observed in N2-like polarized neutrophils (Figure 2A and Supplementary Figure S4). The expression of CEACAM8 (CD66b) and integrin alpha M (CD11b) and the secretion of MPO were markedly increased on N1-like polarized cells as compared to N0 and N2-like cells (Figures 2B–D and Supplementary Figure S3).

These data indicate an activated state of N1-polarized neutrophils as compared to N2-like neutrophils.

### *In vitro* Polarized Neutrophils Show Typical N1 or N2 Cytokine/Chemokine Profiles

**FIGURE 3:**
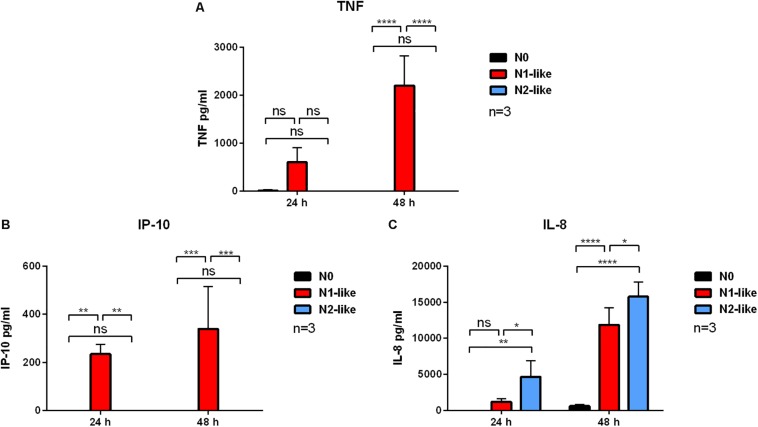
Secretion of tumor necrosis factor (TNF), interferon (IFN)γ-induced protein 10 (IP-10), and interleukin (IL)-8 by N1- and N2-polarized neutrophils. Primary human neutrophils were incubated in the presence of an N1 or N2 polarization cocktail including the Pan-caspase inhibitor QVD-OPh. Neutrophils which were only treated with QVD-OPh (N0) served as control. Cell-free culture supernatants were collected after 24 and 48 h. The concentrations of TNF **(A)**, IP-10 **(B)**, and IL-8 **(C)** were measured by ELISA. Bar diagrams show mean ± SD (*n* = 3). *p < 0.05, **p < 0.01, ***p < 0.001, ****p < 0.0001; ns = not significant.

Next, we assessed the secretion of soluble mediators by *in vitro* polarized neutrophils. Available data from *in vivo* studies indicated an increased release of the pro-inflammatory cytokines TNF and IP-10 (5) by N1 neutrophils. On the contrary, it was shown that N2 neutrophils secrete high amounts of IL-8 (3), which is likely involved in the recruiting of additional neutrophils into the tumor. Cell-free culture supernatants of *in vitro* polarized neutrophils were investigated after 24 and 48 h using ELISA. TNF and IP-10 were only released by N1-like neutrophils; N0 and N2-like polarized neutrophils did not release these pro-inflammatory cytokines (Figures 3A,B). Neutrophils which were cultured with the N2 polarization cocktail for 24 h produced significantly more IL-8 than N1-polarized cells (Figure 3C).

### *In vitro* Polarized N1-Like Neutrophils Show a Tendency to Release More Myeloperoxidase-Derived Reactive Oxygen Species but Less Superoxide Than N2-Like Neutrophils

**FIGURE 4:**
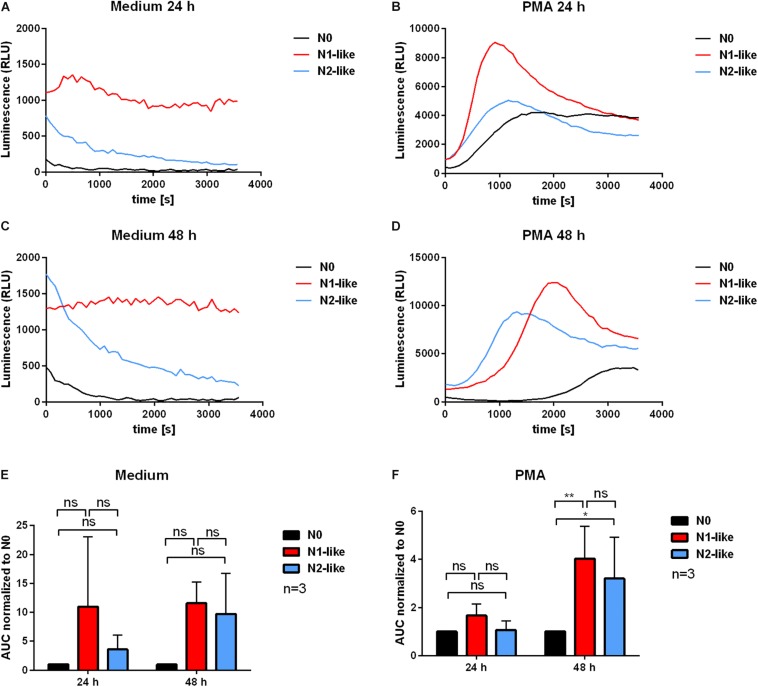
Myeloperoxidase (MPO)-derived reactive oxygen species (ROS) release by *in vitro* polarized neutrophils. Primary human neutrophils were incubated in the presence of an N1 or N2 polarization cocktail including the Pan-caspase inhibitor QVD-OPh. Neutrophils which were only treated with QVD-OPh (N0) served as control. After 24 and 48 h, the MPO-derived ROS release was measured for 1 h at 37°C in medium alone or in the presence or absence of 20 nM phorbol 12-myristate 13-acetate (PMA) by using the luminol-based chemiluminescence assay. Representative curves of luminol chemiluminescence are shown in panels **(A–D)**. Bar diagrams of the area under the curve (AUC) values normalized to N0 (mean ± SD; *n* = 3) are shown in panels **(E)** and **(F)**. *p < 0.05, **p < 0.01, ns = not significant.

**FIGURE 5:**
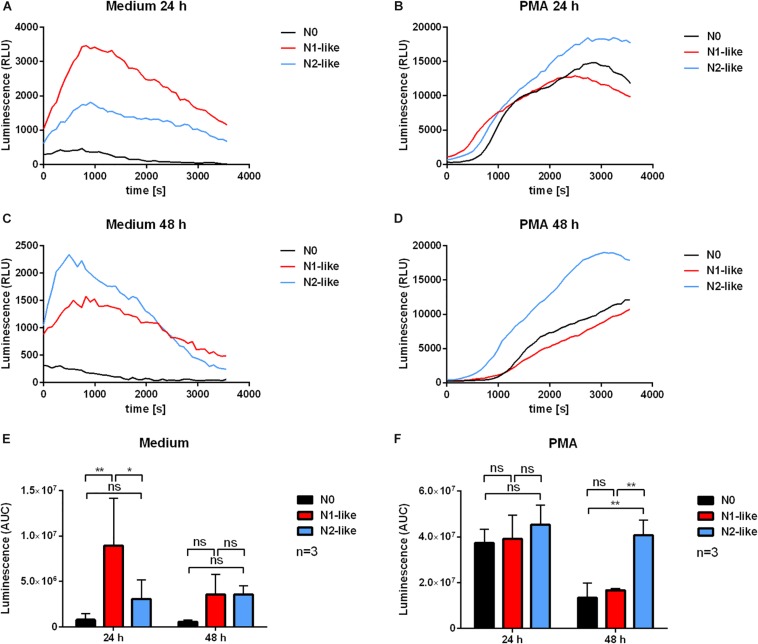
Superoxide release by *in vitro* polarized neutrophils. Primary human neutrophils were incubated in the presence of an N1 or N2 polarization cocktail including the Pan-caspase inhibitor QVD-OPh. Neutrophils which were only treated with QVD-OPh (N0) served as control. After 24 and 48 h, the reactive oxygen species (ROS) release was measured for 1 h at 37°C in medium alone or in the presence of 20 nM phorbol 12-myristate 13-acetate (PMA) by using the lucigenin-based chemiluminescence assay. Representative curves of the time kinetics of lucigenin chemiluminescence are shown in panels **(A–D)**. Bar diagrams of the area under the curve (AUC) values normalized to N0 (mean ± SD; *n* = 3) are shown in panels **(E)** and **(F)**. *p < 0.05, **p < 0.01, ns = not significant.

The role of ROS in the context of TANs is under debate. Although a higher ROS production was reported as a feature of the pro-inflammatory N1 phenotype (6), ROS can have a negative effect on the antitumor defense as well. We assessed the unstimulated and PMA-induced MPO-derived ROS production and extracellular superoxide of *in vitro* polarized neutrophils by the luminol-based and lucigenin-based chemiluminescence assay, respectively. For both time points, 24 and 48 h, N1-polarized neutrophils showed a tendency of higher MPO-derived ROS production compared to N0 and N2-like neutrophils as depicted by the time kinetics of the assay (Figures 4A–D). However, the differences were not statistically significant (Figures 4E,F). Superoxide formation (Figure 5) was significantly increased in N1-polarized neutrophils after 24 h (Figures 5A,E). After 48 h and in the presence of PMA, N2-polarized neutrophils showed a significantly increased superoxide production compared to N0 and N1-polarized cells (Figures 5D,F).

### N2-Polarized Neutrophils Exert Decreased Capacity to Kill *Leishmania donovani* Promastigotes

**FIGURE 6:**
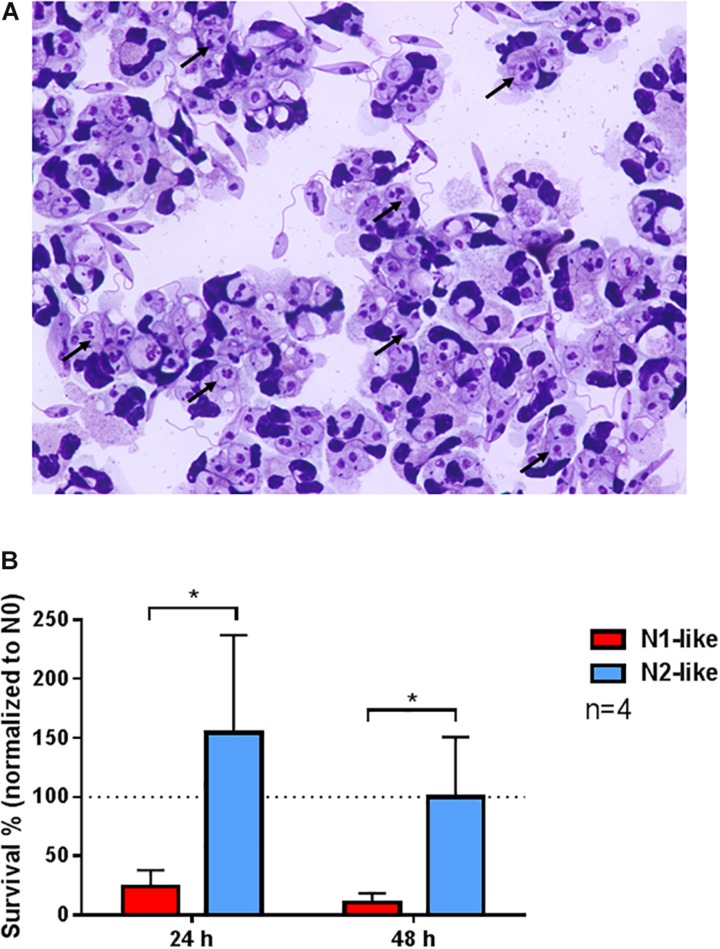
Enhanced survival of *Leishmania donovani* promastigotes in N2-polarized neutrophils *in vitro*. Primary human neutrophils were infected with *L. donovani* promastigotes (ratio 1:10) for 3 h at 37°C, 5% carbon dioxide (CO_2_). Successful infection was determined by Giemsa staining of cytocentrifuged samples, representative microscopy shown in **(A)**. Arrows show representative ingested (intracellular) *Leishmania*. After removing the free, non-ingested parasites, the infected cells were incubated in the presence of an N1 or N2 polarization cocktail at 37°C, 5% CO_2_. Survival of parasites was assessed after 24 and 48 h by the limiting dilution assay **(B)**. Bar diagrams show mean ± SD (*n* = 4). *p < 0.05.

To address our hypothesis that N2-polarized neutrophils are permissive for *L. donovani*, we assessed the anti-leishmanial capacity of *in vitro* polarized neutrophils by a limiting dilution assay. Primary human neutrophils were infected with *L. donovani* promastigotes. The infection rate was nearly 100%, with all neutrophils harboring more than one ingested parasites (Figure 6A). The infected cells were incubated in the presence of an N1 or N2 polarization cocktail for 24 and 48 h, and the parasite load was determined by using a limiting dilution (LD) culture assay. As shown in Figure 6B, N2-like neutrophils harbored significantly more viable parasites as compared to N1-like neutrophils, indicating a decreased killing ability of *L. donovani* promastigotes by N2-like neutrophils.

## Discussion

Equipped with great plasticity, neutrophils can present different phenotypes not only with regard to their stage of differentiation and age but, most importantly, also with regard to their functionality in response to their surrounding microenvironment. The important role of N1 and N2 TANs in the growth and progression of cancer has become clearly evident during the last decade. So far, our current understanding of the N1 and N2 phenotype as well as neutrophil heterogeneity is limited to animal models. Here, we present for the first time a polarization approach to generate N1 and N2 neutrophils *in vitro*. To study long-term polarization effects, primary human neutrophils from healthy donors were incubated up to 48 h in the presence of defined N1 and N2 polarization cocktails containing the caspase inhibitor QVD-OPh to prolong the neutrophil life span. As specific markers for N1 and N2 cells are still missing, we characterized our polarized neutrophils with regard to surface markers, cytokine/chemokine profiles, and effector functions that, based on *in vivo* studies, have been proposed to be characteristic for N1 and N2 neutrophils (Table 2). To our expectation, neutrophils cultured in the presence of N1 polarization cocktail were highly activated, showed a high expression of ICAM-1 and FasR on their surface, and secreted high amounts of IP-10 and TNF. IP-10 (CXCL10) is regarded as a “key driver chemokine” attracting CD8+ and CD4+ T cells to tumor sites and potentiating their function (36). In addition, IP-10 is a major chemokine recruiting natural killer (NK) cells and exerts antitumor effects also by inhibiting angiogenesis (37), making IP-10 a potent antitumor factor (38). Tumor antigen presentation by dendritic cells (DCs) to T cells in lymphoid organs is crucial for induction of antitumor immune responses. Tumor necrosis factor transgene-expressing DCs undergo augmented cellular maturation and induce more robust T-cell activation and antitumor immunity than DCs generated in recombinant TNF (39). Importantly, IP-10 and TNF were shown to act in synergy to induce antitumor effects (40).

Neutrophils treated with the N2 polarization cocktail were less activated than N1-like neutrophils and showed lower L-selectin shedding. Further, they expressed the typical N2 receptor CXCR2 to a high extent on their surface and secreted elevated amounts of IL-8. The IL-8/IL-8R axis is a signaling pathway used by tumor cells to recruit neutrophils initially to the tumor site. As N2 neutrophils are a potent source of IL-8, the entrapping influx of neutrophils into the tumor is established as a positive feedback loop (41).

As an important effector function of neutrophils, we measured ROS release of *in vitro* polarized cells. Our findings show a tendency of N1-like neutrophils for a higher MPO-derived ROS release than N2-like neutrophils. However, these differences were statistically not significant. Also the assessment of superoxide production produced inconsistent results being higher in N1-like cells after 24 h but being higher in PMA-treated N2-like cells after 48 h of culture. These findings may reflect the two-faced role of ROS in tumor biology. On one hand, ROS can induce oxidative damage on tumor cells (42). On the other hand, neutrophil-derived ROS may be mutagenic and contribute to genomic instability. In addition, both ROS-dependent and -independent processes for neutrophil antibody-dependent cell cytotoxicity have been suggested (43). Although neutrophil-derived ROS might damage and destroy tumor cells, they can also cause genotoxicity and act in an immunosuppressive manner by reducing the expression of the T cell receptor CD3 ζ chain (44).

In previous studies, we demonstrated that *Leishmania* uses neutrophils as a “Trojan horse” to enter their definite host cells, macrophages (45). Despite numerous effector mechanisms, it seems that the anti-leishmanial capacities of neutrophils are not always sufficient to eliminate the parasite. Here, we hypothesized that neutrophils are polarized during *L. donovani* infection toward an N2-like phenotype which is permissive for the parasite and favors the parasite’s survival. Our results showed that *in vitro* polarized N2-like neutrophils exert a decreased killing ability for *L. donovani* promastigotes compared to N1-like neutrophils which eliminated the majority of parasites. As neutrophils are the first innate immune cells at the site of infection, it would be of advantage for the parasite to polarize the neutrophils toward a favorable phenotype. Although our results suggest that the presence of pro- and anti-inflammatory neutrophil phenotypes can modulate the outcome of VL, they must be interpreted with caution as our polarization cocktail is based on conditions present in the tumor microenvironment. However, several factors that are characteristic for the tumor microenvironment such as hypoxia (46, 47) or the cytokines IL-10 (48, 49), TGF-β (50, 51), and the lipid mediator PGE_2_ (48, 49) are also found in tissues infected with *Leishmania*.

Our study revealed that it is feasible to polarize mature human neutrophils *in vitro* to distinct phenotypes. We could show functional and phenotypical differences between neutrophils cultured in the presence of N1- or N2-polarizing cocktails. Our findings indicate that even fully differentiated mature neutrophils maintain their plasticity and can acquire typical phenotypic characteristics observed so far only in the tumor microenvironment. Our novel *in vitro* model for the polarization of N1- and N2-like neutrophils represents a first step, allowing the study of pro- and anti-inflammatory neutrophils. Investigation of *in vitro* polarized pro- and anti-inflammatory neutrophil populations could be a major advantage for the identification of biomarkers for tumor-promoting neutrophils and could contribute to the development of therapeutic strategies to target disease-promoting neutrophils.

## Data Availability Statement

The raw data supporting the conclusion of this article will be made available by the authors, without undue reservation, to any qualified researcher.

## Ethics Statement

The studies involving human participants were reviewed and approved by the Ethical Committee of the University of Lübeck. The patients/participants provided their written informed consent to participate in this study.

## Author Contributions

MO designed the study, carried out the experiments, and wrote the manuscript. SM carried out the experiments. TL designed the study and wrote the manuscript.

## Conflict of Interest

The authors declare that the research was conducted in the absence of any commercial or financial relationships that could be construed as a potential conflict of interest.

## Abbreviations

ANCA: anti-neutrophil cytoplasmic antibodies
ANOVA: analysis of variance
APC: allophycocyanin
AUC: area under the curve
ELISA: enzyme-linked immunosorbent assay
FACS: fluorescence-activated cell sorting
FCS: fetal calf serum
FITC: fluorescein isothiocyanate
G-CSF: granulocyte-colony stimulating factor
HIV: human immunodeficiency virus
ICAM-1: intercellular adhesion molecule 1
IFN: interferon
IgG: immunglobulin G
IgM: immunglobulin M
IL: interleukin
LPS: lipopolysaccharide
mAb: monoclonal antibody
MMP: matrix metalloproteinase
NETs: neutrophil extracellular traps
PE: phycoerythrin
PMA: phorbol-12-myristate-13-acetate
PMN: polymorphonuclear leukocytes
ROS: reactive oxygen species
SLE: systemic lupus erythematosus
TAMs: tumor-associated macrophages
TANs: tumor-associated neutrophils
TGF- β: transforming growth factor beta
TNF: tumor necrosis factor
VEGF: vascular endothelial growth factor
VL: visceral leishmaniasis.
