# Supplementary material for: An Attempt to Polarize Human Neutrophils Toward N1 and N2 Phenotypes in vitro
Source: Front Immunol. 2020 Apr 28;11:532. doi: 10.3389/fimmu.2020.00532 (PMC7198726; doi:10.3389/fimmu.2020.00532)
Supplement: Supplementary file 1 [file Data_Sheet_1.pdf]

Supplementary Material

Supplementary Figure 1

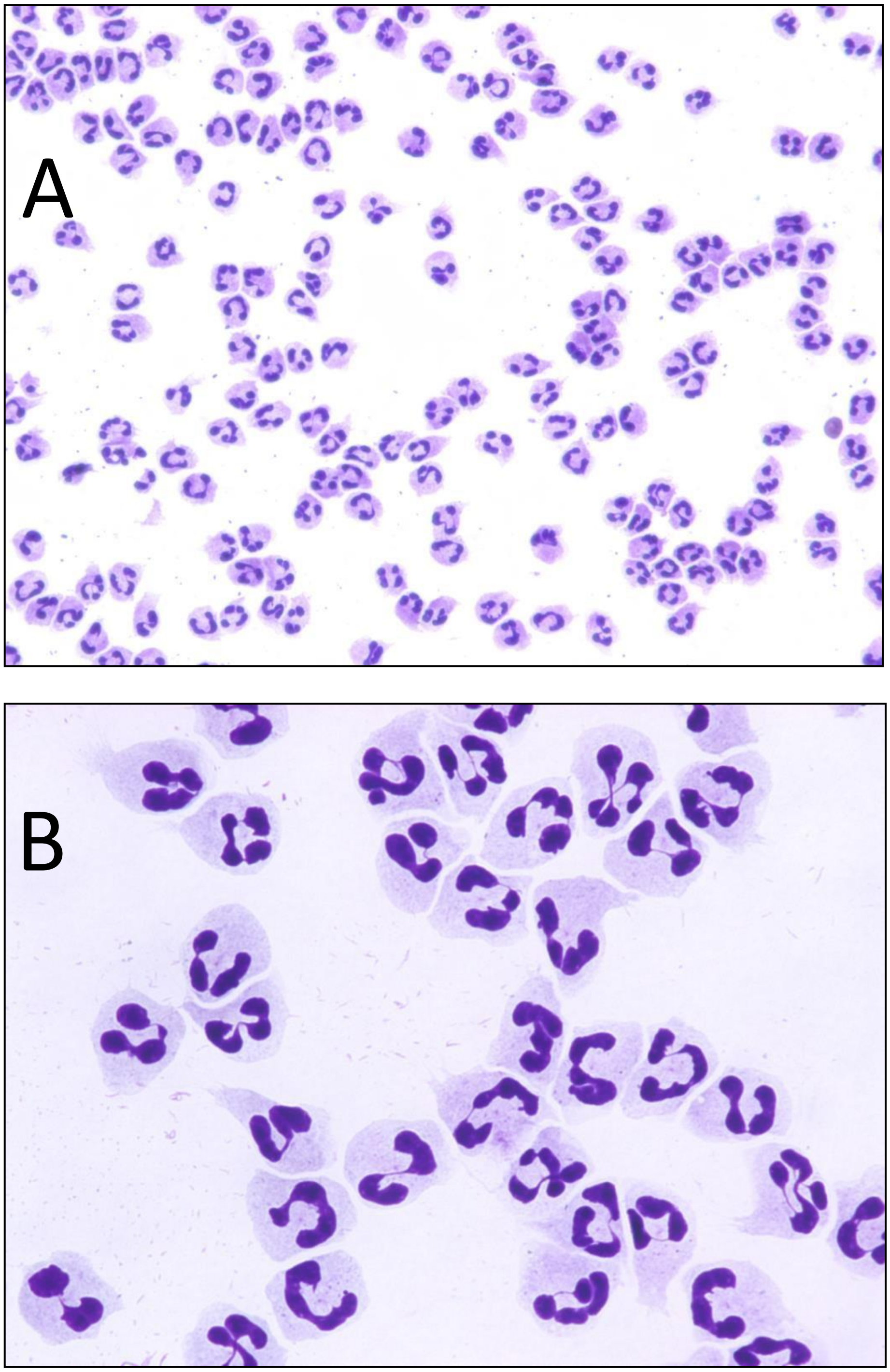

Supplementary Figure 1. **Microphotographs illustrating the purity of neutrophil preparations.**

Neutrophils were isolated from peripheral blood of healthy volunteers as described in **Materials and Methods**. Cytocentrifuge preparates were stained with Diff Quik Fix (Medion Diagnostics, Berlin, Germany) and visualized with a Keyence BZ-9000 (Keyence, Osaka, Japan) light microscope. **A** 40x magnification, **B** 100x magnification,

Supplementary Figure 2

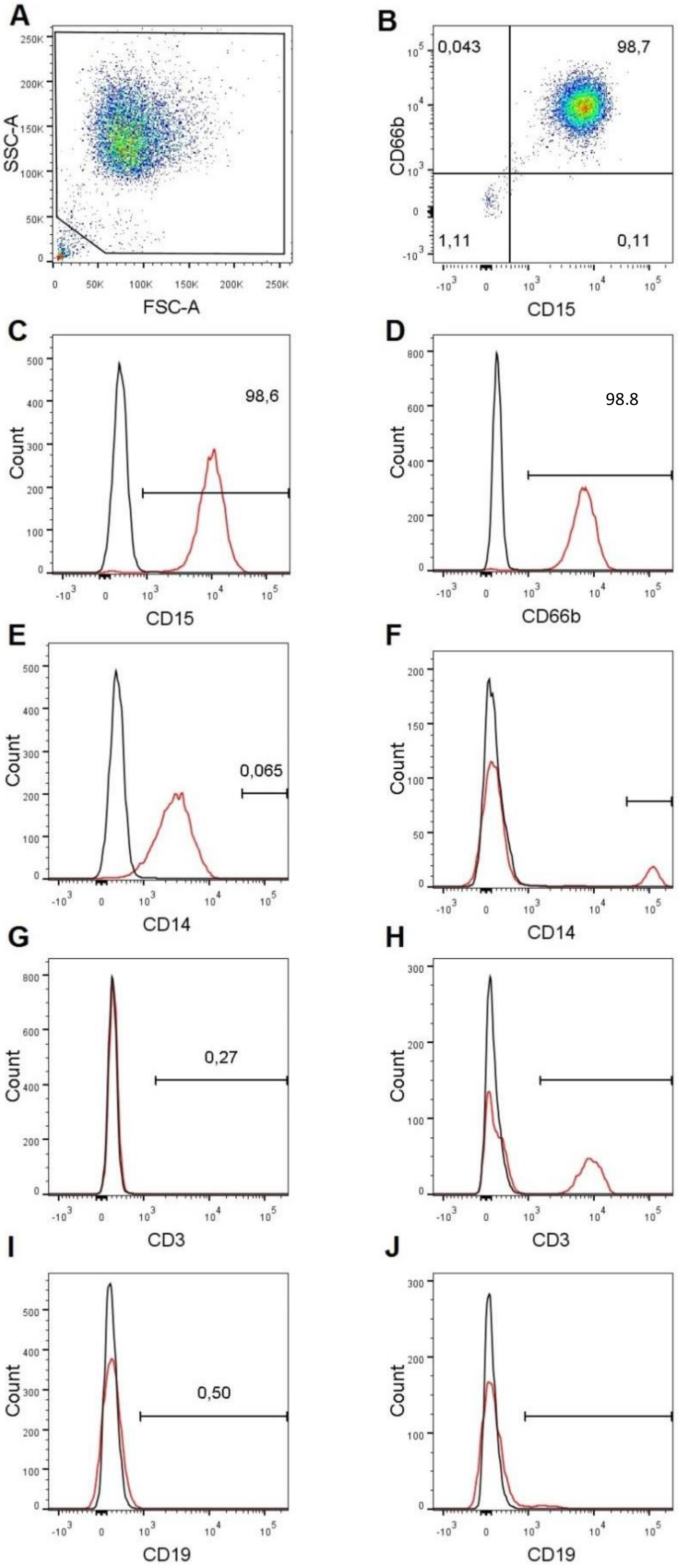

Supplementary Figure 2. **Analysis of neutrophil purity by flow cytometry.**

Neutrophils were isolated from peripheral blood of healthy volunteers as described in **Materials and Methods**. **A**) Forward-scatter vs. side-scatter dot plot of neutrophils showing the applied gating strategy for the isolated neutrophils. **B)** Dot plot of isolated cells stained with PE-conjugated mAb to human CD15 (clone 80H5, IgM, Immunotech, Marseille, France) and FITC-conjugated mAb to human CD66b (clone G10F5, IgM, BD, Heidelberg, Germany). Histograms of isolated neutrophils stained with **C**) anti-CD66b or **D**) anti-CD15 antibody.

Representative histograms of the isolated neutrophil population **(E)** or PBMCs **(F)** as control for the monocyte-specific marker CD14 (clone TÜK4, IgG2a, Dako, Waldbronn, Germany). Representative histograms of the isolated neutrophil population **(G)** or PBMCs **(H)** as control for the T-cell-specific marker CD3 (clone REA613, IgG1, Bergisch Gladbach, Germany). Representative histograms of the isolated neutrophil population **(I)** or PBMCs **(J)** as control for the B-cell-specific marker CD19 (clone 4G7-2E3, IgG1, R&D Systems, Wiesbaden, Germany).

Black lines in the histograms represent unstained cells and red lines represent cells stained with the respective fluorochrome-conjugated antibody. The numbers indicate the % of the cells in the given quadrant or region.

Supplementary Figure 3

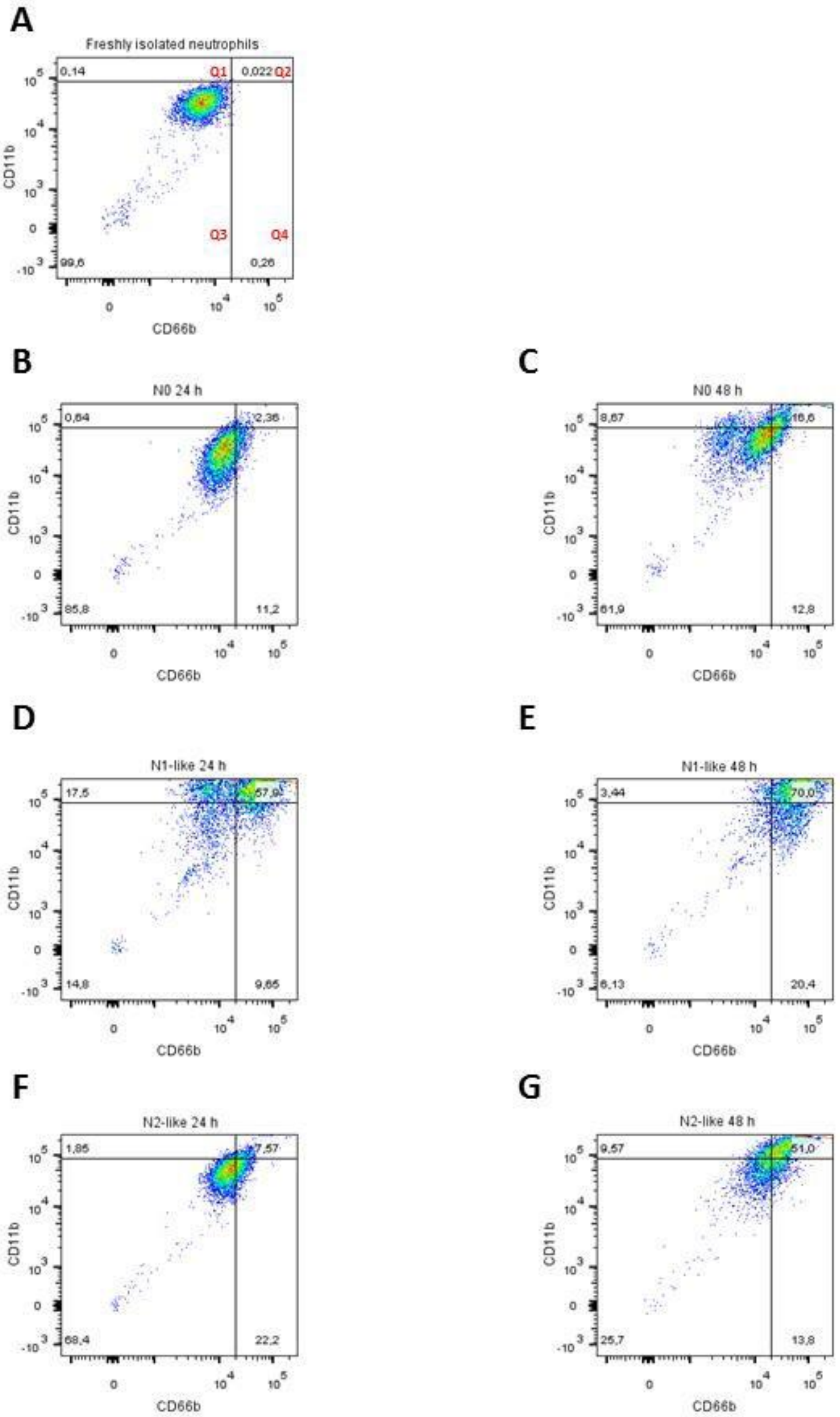

Supplementary Figure 3. Expression of the activation markers CD11b and CD66b by *in vitro* polarized neutrophils.

Primary human neutrophils were incubated for 24 h or 48 h in the presence of a N1 or N2 polarization cocktail containing the Pan-caspase inhibitor QVD-OPh. Neutrophils which were only treated with QVD-OPh (N0) served as control. Representative dot plots of the cell surface expression of CD66b and CD11b assessed by flow cytometry.

A) Freshly isolated neutrophils. The quadrants were set to define the CD66b^{high} cells (cells in the Q2 + Q4 quadrants) and CD11b^{high} cells (cells in the Q1 + Q2 quadrants). Dot plots in **B, D** and **F** show the expression of CD66b and CD11b of N0, N1-like and N2-like neutrophils, respectively, after 24 h of culture. Dot plots in **C, E** and **G** show the expression of CD66b and CD11b of N0, N1-like and N2-like neutrophils, respectively, after 48 h of culture. The numbers indicate the ratio (%) of the cells in the given quadrant.

Supplementary Figure 4

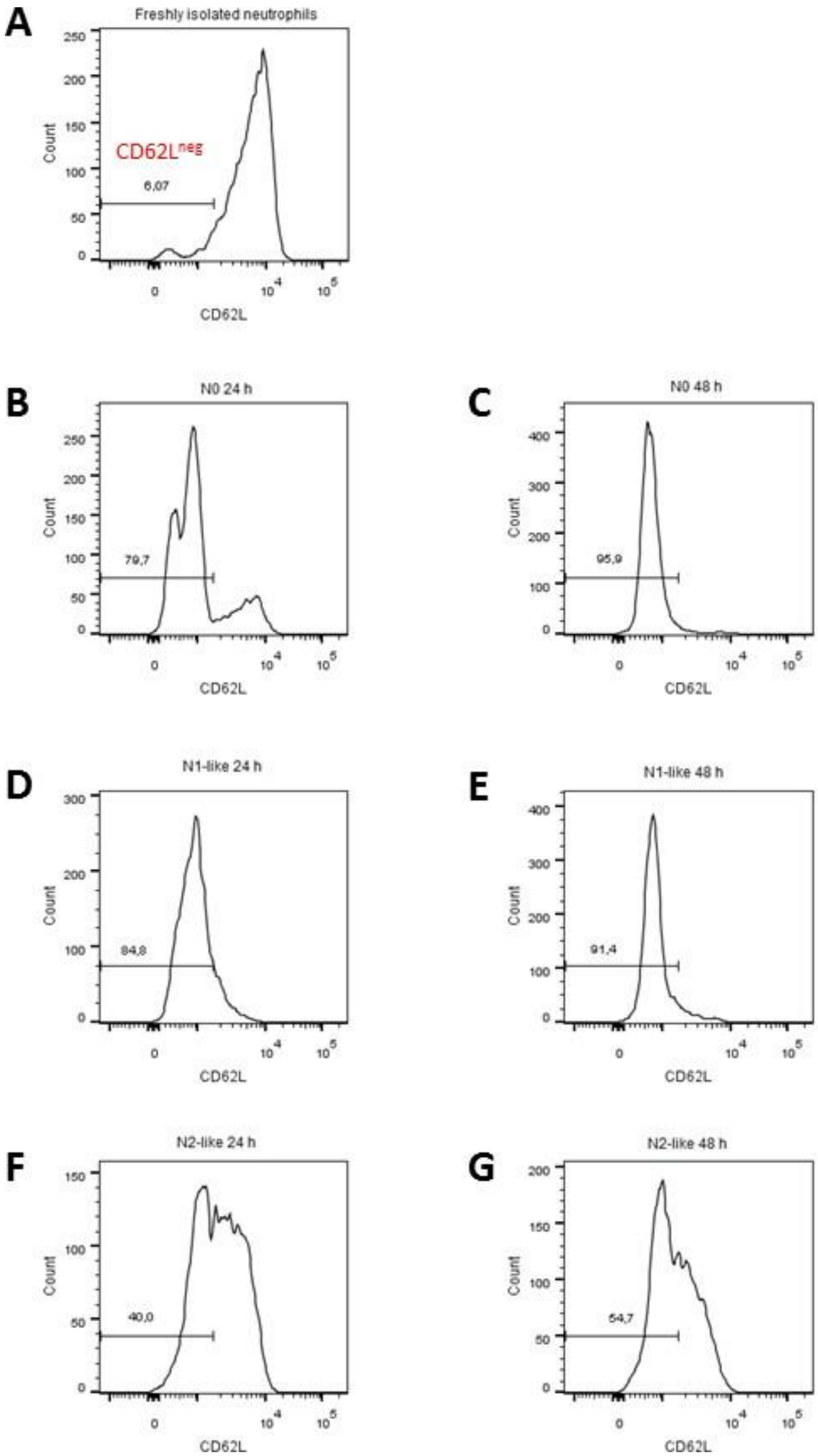

41

Supplementary Figure 4. Expression of the activation marker CD62L by *in vitro* polarized neutrophils.

Primary human neutrophils were incubated for 24 h or 48 h in the presence of a N1 or N2 polarization cocktail containing the Pan-caspase inhibitor QVD-OPh. Neutrophils which were only treated with QVD-OPh (N0) served as control. Representative histograms of the cell surface expression of CD62L assessed by flow cytometry.

A) Freshly isolated neutrophils. The region was set to define the CD62L^{neg} cells. Histograms in **B, D** and **F** show the CD62L expression of N0, N1-like and N2-like neutrophils, respectively, after 24 h of culture. Dot plots in **C, E** and **G** show the CD62L expression of CD62L of N0, N1-like and N2-like neutrophils, respectively, after 48 h of culture. The numbers indicate the ratio (%) of CD62L^{neg} cells.

Supplementary Figure 5

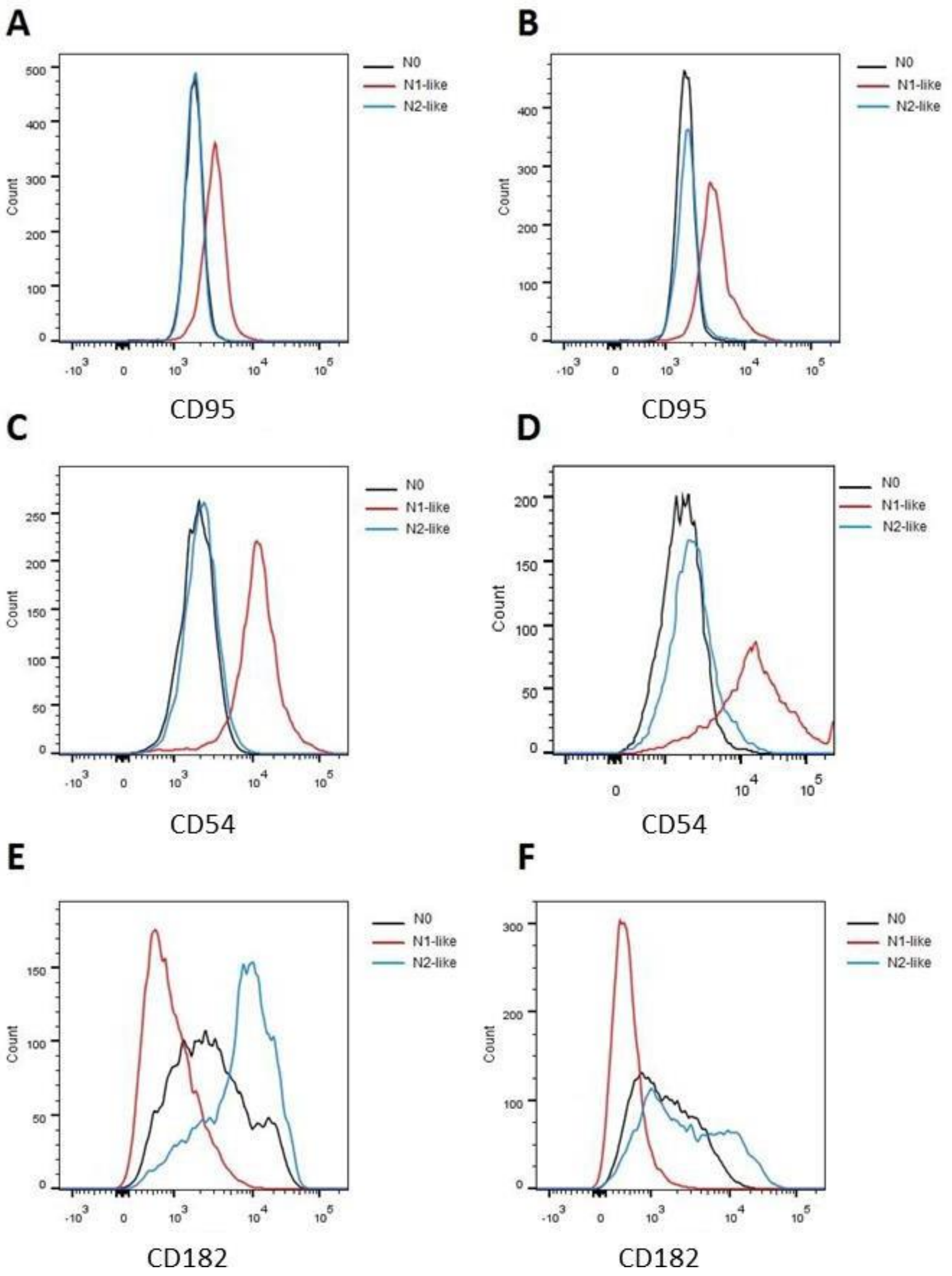

Supplementary Figure 5. Cell surface expression of typical N1 and N2 markers on *in vitro* polarized neutrophils.

Primary human neutrophils were incubated for 24 h or 48 h in the presence of a N1 or N2 polarization cocktail containing the Pan-caspase inhibitor QVD-OPh. Neutrophils which were only treated with QVD-OPh (N0) served as control. The cell surface expression of the typical N1 markers FasR (CD95) (**A, B**) and ICAM-1 (CD54) (**C, D**) as well as the typical N2 marker CXCR2 (CD182) (**E, F**) was assessed by using flow cytometry. Representative histograms of polarized neutrophils stained after 24 h **(A, C, E)** and 48 h **(B, D, F)** of culture.

Supplementary Figure 6

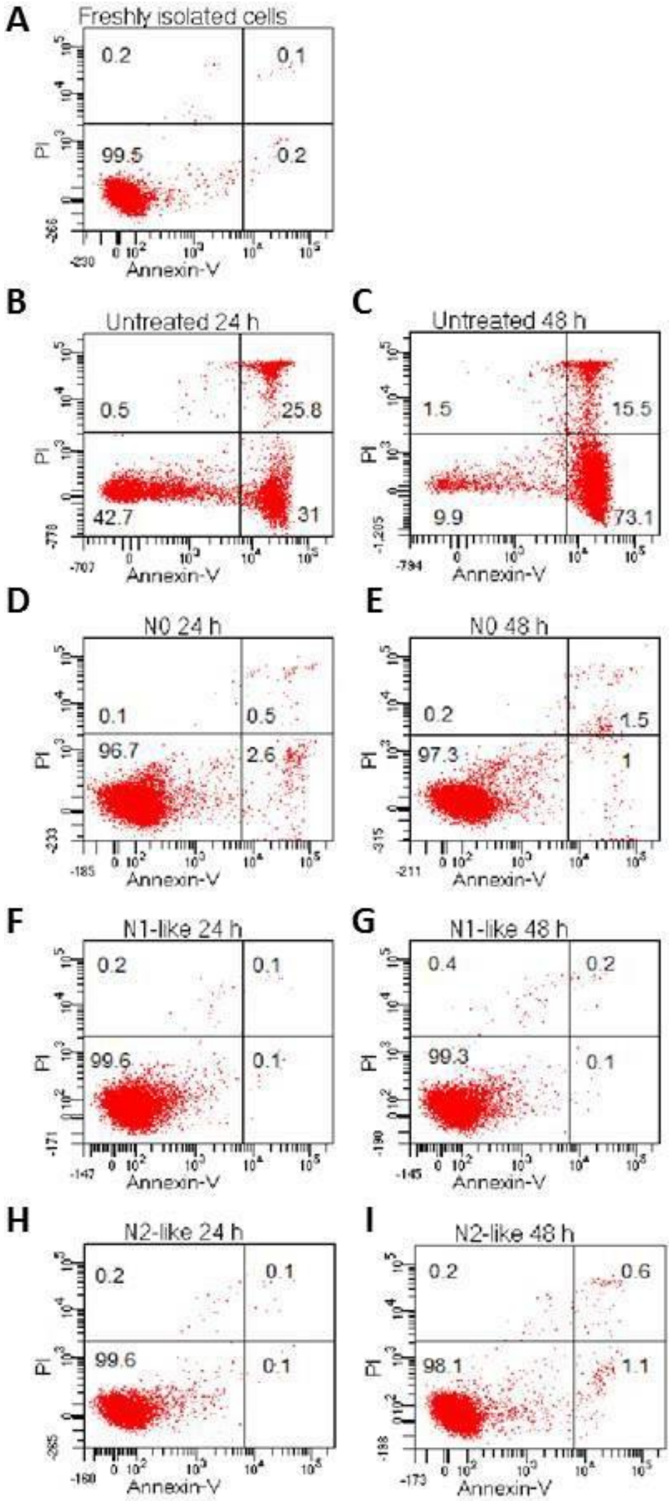

Supplementary Figure 6. Apoptosis and viability of neutrophils cultured in the absence or presence of the caspase inhibitor QVD-OPh.

Primary human neutrophils were treated with 3 µM QVD-OPh and incubated for 24 h and 48 h. Apoptosis and cell viability was assessed by flow cytometry of cells stained with Annexin-V and PI and analyzed by flow cytometry. **A)** Freshly isolated neutrophils. **B-C)** Neutrophils cultured in the absence of QVD-OPh for 24 h and 48 h, respectively. **D-E)** Neutrophils cultured without polarization cocktail in the presence of QVD-OPh for 24 h and 48 h, respectively. **F-G)** Neutrophils cultured with N1 polarization cocktail in the presence of QVD-OPh for 24 h and 48 h, respectively. **H-I)** Neutrophils cultured with N2 polarization cocktail in the presence of QVD-OPh for 24 h and 48 h, respectively. The numbers indicate the ratio (%) of the cells in the given quadrant.

Supplementary Figure 7

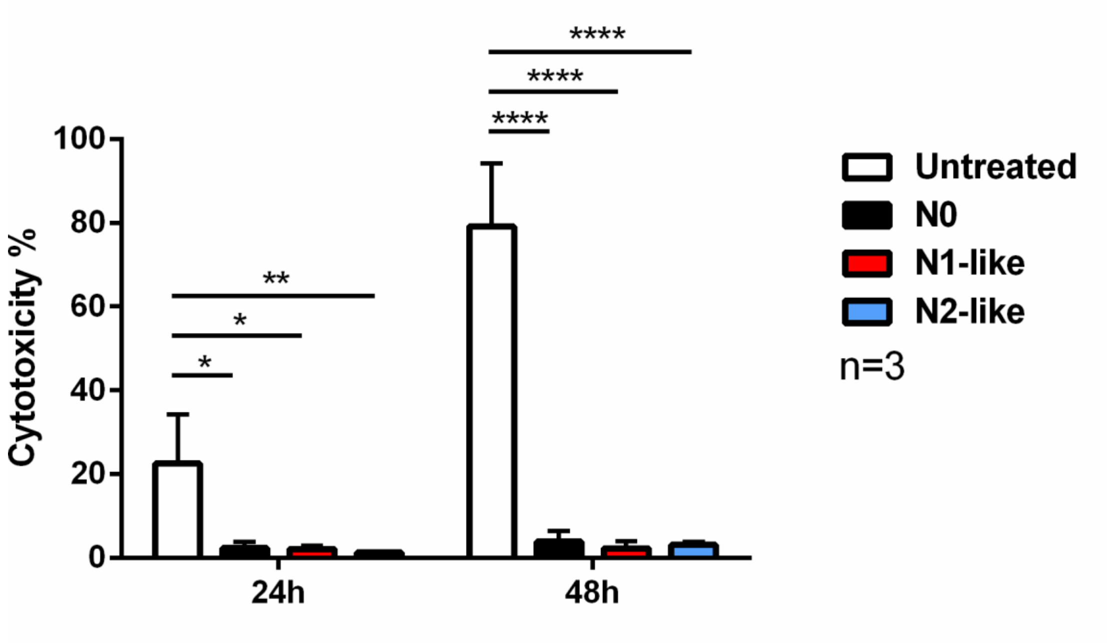

Supplementary Figure 7. Release of LDH by neutrophils cultured in the absence or presence of the caspase inhibitor QVD-OPh

Primary human neutrophils were incubated in the presence of a N1 or N2 polarization cocktail including the Pan-caspase inhibitor QVD-OPh. Neutrophils which were only treated with QVD-OPh (N0) served as control. Untreated cells were cultured in the absence of QVD-OPh. Released lactate dehydrogenase (LDH) was measured in cell-free culture supernatants after 24 h and 48 h by using the CytoTox 96® Non-Radioactive Cytotoxicity Assay (Promega) in accordance with the manufacturer's instructions. The bar diagram shows mean cytotoxicity (% of total lysis) ± SD (n=3). 4 *p<0.05, **p<0.01, ****p<0.0001.

Supplementary Figure 8

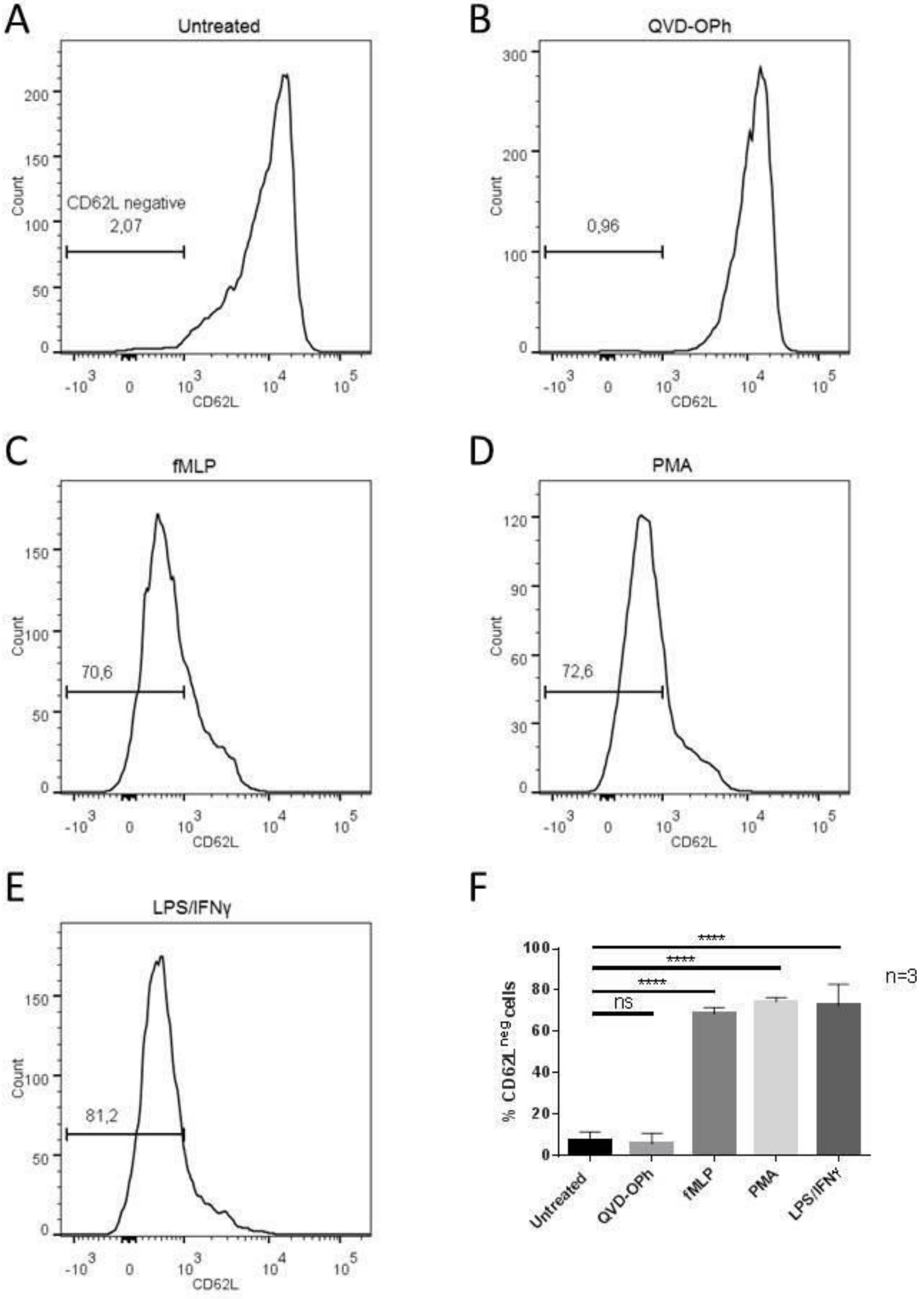

Supplementary Figure 8. Exposure to QVD-OPh does not activate primary human neutrophils

Primary human neutrophils were incubated for 60 min in the presence of 3 μM QVD-OPh. As positive controls for neutrophil activation 1 μM fMLP, 20 nM PMA and 100 ng/ml LPS + 200 U/ml IFNγ were used. The cell surface expression of CD62L was assessed by flow cytometry. Representative histograms of **A)** untreated neutrophils and neutrophils treated with **B)** QVD-OPh, **C)** fMLP, **D)** PMA and **E)** LPS + IFNγ. The numbers indicate the ratio (%) of $CD62L^{neg}$ cells. The bar diagram (**F)** shows mean ratio (%) of $CD62L^{neg}$ cell ± SD (n=3). ***p<0.001, ****p<0.0001, ns = not significant

Supplementary Figure 9

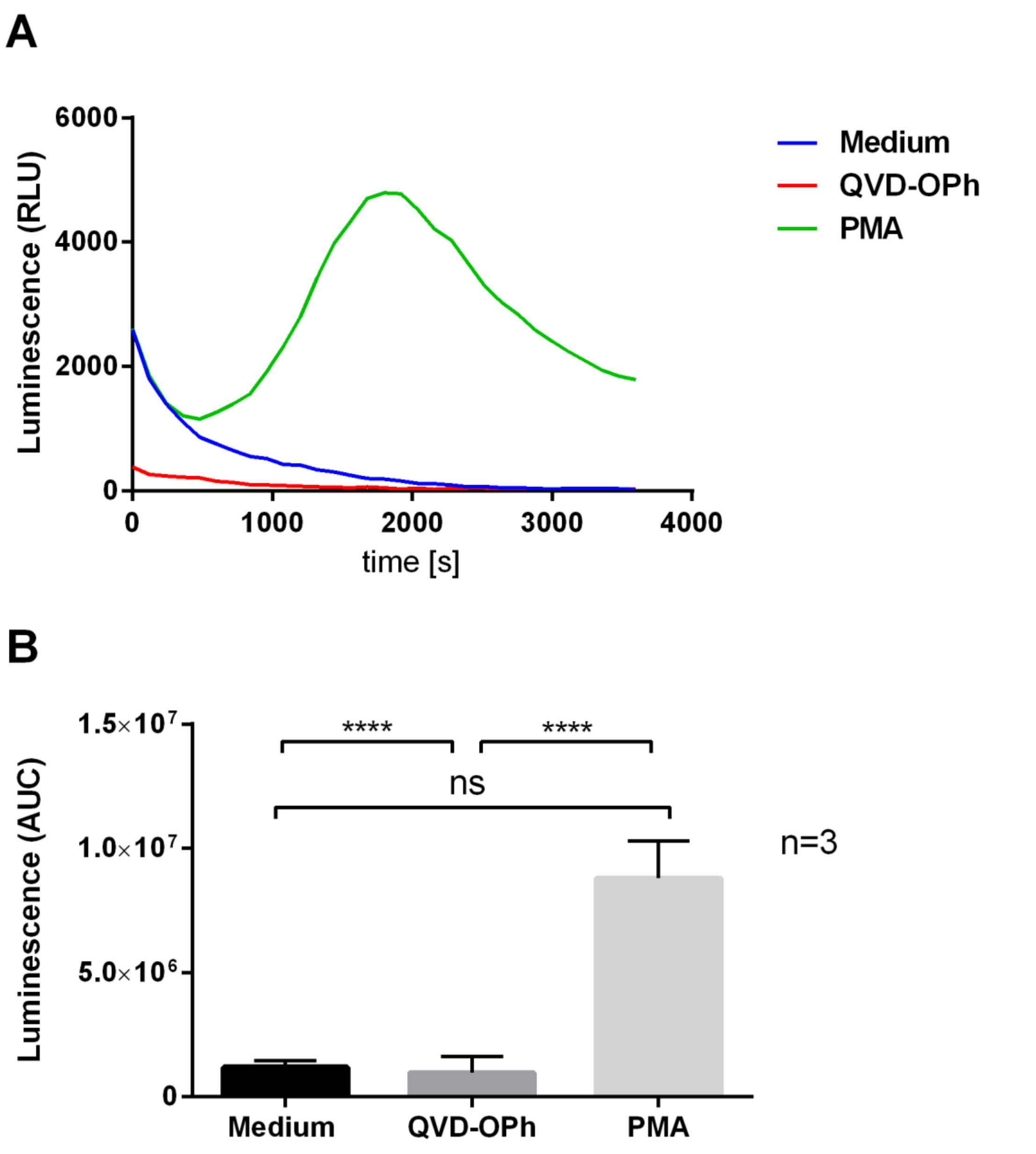

Supplementary Figure 9. Exposure to QVD-OPh does not induce ROS release by primary human neutrophils.

Primary human neutrophils were incubated for in the presence of 3 μM QVD-OPh. PMA (20 nM) was used as positive control for the induction of ROS production. The ROS release was measured for 1 h at 37°C in medium alone or in the presence of QVD-OPh or PMA by using the luminol-based chemiluminescence assay. **A)** Representative time kinetics curve of the of luminol chemiluminescence. **B**) Bar diagrams of the area under the curve (AUC) values (mean ± SD; n=3). ****p<0.0001, ns = not significant
